# Association of human papilloma virus (HPV) infection with oncological outcomes in urothelial bladder cancer

**DOI:** 10.1186/s13027-020-00318-3

**Published:** 2020-08-28

**Authors:** Solmaz Ohadian Moghadam, Kamyar Mansori, Mohammad Reza Nowroozi, Davoud Afshar, Behzad Abbasi, Ali Nowroozi

**Affiliations:** 1 Uro-Oncology Research Center, Tehran University of Medical Sciences, Keshavarz Blvd, Tehran, 1419733141 Iran; 2 Department of Epidemiology and Biostatistics, School of Medicine, Zanjan University of Medical Sciences, Zanjan, Iran; 3 Department of Microbiology and Virology, School of Medicine, Zanjan University of Medical Sciences, Zanjan, Iran

**Keywords:** Bladder cancer, Transitional cell carcinoma (TCC), Human papilloma virus (HPV), Tumor grade, Tumor stage, Recurrence

## Abstract

**Background:**

Bladder cancer is one of the leading causes of cancer death in adults worldwide. There are various risk factors described for the bladder cancer development including genetic background as well as environmental exposure. Currently, infectious agents such as human papilloma virus (HPV) has also been linked to bladder cancer risk. The current study aimed to evaluate the potential correlation between HPV infection and the oncological outcome in urothelial bladder cancer.

**Methods:**

Totally 106 tissue samples of histopathologically confirmed transitional cell carcinoma (TCC) of the urinary bladder were included in this study. The presence of high risk (types 16 and 18) and low risk (types 11 and 6) types of HPV was evaluated using polymerase chain reaction (PCR) followed by in situ hybridization.

**Results:**

Out of 106 bladder cancer patients, a total of 24 cases (22.6%) were positive HPV infection. The most common type of HPV detected was type 16 followed by types 11 and 18, and 6. According to independent T-test results, there was a significant association between mean age and HPV infection (*P* = 0.015). Moreover, our findings showed a significant relation between infection with HPV and tumor stage, tumor grade, muscle invasion of the tumor, as well as tumor recurrence. The results of Chi-square Test indicated that there is significant statistical association between types of HPV and tumor grade (*P*-Value = 0.044).

**Conclusion:**

Our findings indicated that a family history of cancer and HPV infection can be potential independent predictive factors for tumor recurrence in bladder cancer. Overall, the results of this study strongly indicate a significant relationship between HPV infection and an aggravated outcome of the disease and a higher risk of recurrence in patients with bladder cancer.

## Background

Bladder cancer is one of the leading causes of cancer death in adults worldwide and approximately 400,000 new cases and 186,000 deaths annually has been reported globally [1]. There are two significant entities for bladder cancer including low grade superficial tumors and high grade invasive tumors [2]. Transitional cell carcinoma (TCC) is the most frequent pathological subtype of bladder cancer, consisting of > 90% of all cases [2]. Several risk factors recognized to be involved in the etiology of bladder cancer, including cigarette smoking, exposure to chemicals such as aromatic amines and 4,4′-methylenebis, and schistosomiasis in some regions [3]. Moreover, the infectious agents such as human papilloma virus (HPV) also has been suggested to be involved in human tumorigenesis [4]. Studies have shown a role for viruses in 15 to 20% of all human cancer cases [5] and nearly 10% of the global cancer burden is associated to HPV infection [6]. HPV is one of the major causes of viral sexually transmitted infections (STIs) and has been suggested as a potential risk factor for development of genitourinary cancers [7]. The etiologic role of high risk types of HPV (16 and 18) in development of different cancers has been suggested previously [8–11]. Currently, there are several studies that described a correlation between HPV and development of bladder cancer. However, their found contradictory results [12–14].

At present, avoiding risk factors is the only way to prevent bladder cancer and the treatments are invasive and costly [15]. Thus, appropriate predictive and prognostic factors for therapy response may improve the treatment outcomes. Therefore, study on HPV as an infectious agent associated in the etiology of bladder cancer might affect prevention and therapy [15].

In the present study, we have evaluated the presence of high risk (types 16 and 18) and low risk (types 6 and 11) HPV types in bladder tumors and the possible correlation between HPV infection and oncological outcomes of the disease.

## Methods

### Study subjects

A total of 106 formalin fixed-paraffin embedded (FFPE) tissue samples from patients with histopathologically confirmed TCC of the urinary bladder were enrolled in this study. A self-administered questionnaire for clinicopathologic characteristics and demographic data was completed by the patients. Grading and staging were carried out according to the American Joint Committee on Cancer’s 2002 TNM staging system and World Health Organization (WHO) respectively. Individuals with the history of other malignancies and/or cancer metastasized to bladder from another origin were excluded. This study was approved by the institutional Ethics Committee.

### HPV detection

Genomic DNA was extracted from tissue samples by QIAamp DNA FFPE Tissue kit (Qiagen GmbH, Hilden, Germany) according to the manufacturer’s guideline. The tissue samples subjected to the amplification of a highly conserved late region I (L1) of HPV genome by PCR assay using consensus primer pairs (MY09/MY11, GP5+/6+) to detect all recognized HPV types, as described previously [16]. The amplification of β-globin gene was carried out as internal control [17]. Subsequently, in situ hybridization was accomplished for typing of HPV according to the procedure which was described in detail previously [17].

### Statistical analysis

In this study, descriptive statistics (frequency, mean and standard deviation) and inferential statistical tests (independent t-test, analysis of variance (ANOVA), Chi-square and Fisher’s exact test) were used.

The relationship between tumor grade, tumor stage, as well as recurrence after 18 month, and smoking status, family history of cancer, and HPV infection was assessed using crude and age-sex adjusted of odds ratio and their 95% confidence intervals by logistic and penalized logistic regression. All analysis performed by STATA software ver. 13.

## Results

Categorical variables and their distributions in each group was compared by chi-squared or Fisher’s exact test and continuous variable (age) was compared using independent t-test.

In the present study, out of 106 bladder cancer patients examined, a total of 24 cases (22.6%) were positive HPV infection. The most common type of HPV detected was type 16 with a frequency of 10 (9.4%); followed by types 11 and 18, and 6 with frequencies of 5.7, 5.7, and 0.9%, respectively. The mean age of the subjects was 62.98 ± 10.26 years (rang from 38 to 89 years). The mean age of patients infected with HPV was 58.54 ± 11.39 years. The mean age of patients who were negative for HPV infection was 64.28 ± 9.59 years. According to independent T-test results, there was a significant association between mean age and HPV infection (*P* = 0.015). The association between patient characteristics and HPV status were presented in Table 1. Our findings showed a significant relation between infection with HPV and tumor stage, tumor grade, muscle invasion of the tumor, as well as tumor recurrence (Table 1).

**Table 1:** The association between patient characteristics and HPV status of patients

	Group	HPV		***p***-value
		Negative (%)	Positive (%)	
**Sex**	Male	64 (78)	21 (87.5)	0.393^a^
	Female	18 (22)	3 (12.5)	
**Cigarette smoker**	Smoker	59 (72)	22 (91.7)	**0.045**^**b**^
	Non-smoker	23 (28)	2 (8.3)	
**Family history of cancer**	Positive	36 (43.9)	11 (45.8)	0.867^b^
	Negative	46 (56.1)	13 (54.2)	
**Tumor grade**	Low grade	52 (63.4)	7 (29.2)	**0.003**^**b**^
	High grade	30 (36.6)	17 (70.8)	
**Tumor stage**	Ta	45 (54.9)	5 (20.8)	**< 0.0001**^**a**^
	T1	10 (12.2)	3 (12.5)	
	T2	26 (31.7)	7 (29.2)	
	T3-T4	1 (1.2)	9 (37.5)	
**Muscle invasion**	Positive	27 (32.9)	16 (66.7)	**0.003**^**b**^
	Negative	55 (67.1)	8 (33.3)	
**Recurrence after 18 month**	Positive	37 (45.1)	24 (100)	**< 0.0001**^**b**^
	Negative	45 (54.9)	0 (0)	
^a^ Fisher exact test; ^b^ Chi square test				

Table 2 demonstrates the association between patient characteristics and oncological outcomes (tumor grade, tumor stage, muscle invasion, and recurrence after 18 month). As can be seen, there were significant statistical association between age and tumor stage (*P* = 0.005) and recurrence after 18 month (*P* = 0.015). Significant statistical association was also observed between family history of cancer and tumor grade (*P* = 0.042), muscle invasion (*P* = 0.018), and recurrence after 18 month (*P* < 0.001).

**Table 2:** The association between patient characteristics and tumor grade, tumor stage, muscle invasion, and recurrence after 18 month

Variable	Age (year)	Sex		Smoking		Family history of cancer	
	Mean (SD)	Male (%)	Female (%)	Yes (%)	No (%)	Positive (%)	Negative (%)
Tumor Grade							
**Low grade**	62.71 (10.45)	46 (54.10)	13 (61.90)	44 (54.3)	15 (60)	21 (44.70)	38 (64.40)
**High grade**	63.32 (10.13)	39 (45.90)	8 (38.10)	37 (45.7)	10 (40)	26 (55.30)	21 (35.60)
*P*-Value	0.764 ^**a**^	0.520 ^**c**^		0.617 ^**c**^		**0.042** ^**c**^	
Tumor Stage							
**Ta**	62.02 (9.99)	40 (47.10)	10 (47.60)	38 (46.90)	12 (48.00)	17 (36.20)	33 (55.90)
**T1**	67.92 (12.57)	10 (11.80)	3 (14.30)	10 (12.30)	3 (12.00)	5 (10.60)	8 (13.60)
**T2**	65.15 (8.39)	26 (30.60)	7 (33.30)	24 (29.60)	9 (36.00)	21 (44.70)	12 (20.30)
**T3-T4**	54.20 (9.00)	9 (10.60)	1 (4.80)	9 (11.10)	1 (4.00)	4 (8.50)	6 (10.20)
*P*-Value	**0.005** ^**b**^	0.865 ^**c**^		0.734 ^**c**^		0.060 ^**c**^	
Muscle Invasion							
**Positive**	62.60 (9.64)	35 (41.20)	8 (38.10)	33 (40.70)	10 (40)	25 (53.20)	18 (30.50)
**Negative**	63.24 (10.73)	50 (58.80)	13 (61.90)	48 (59.30)	15 (60)	22 (46.80)	41 (69.50)
*P*-Value	0.757 ^**a**^	0.797 ^**c**^		0.947 ^**c**^		**0.018** ^**c**^	
Recurrence after 18 months							
**Positive**	60.92 (10.63)	49 (57.60)	12 (57.10)	49 (60.50)	12 (48.00)	36 (76.6)	25 (42.40)
**Negative**	65.78 (9.13)	36 (42.40)	8 (42.90)	32 (39.50)	13 (52.00)	11 (23.4)	34 (57.60)
*P*-Value	**0.015** ^**a**^	0.976 ^**c**^		0.296 ^**c**^		**< 0.001** ^**c**^	

^a^ Independent t test; ^b^ Analysis of variance (ANOVA); ^c^ Chi-square test

The association between types of HPV and sex, tumor grade, tumor stage, muscle invasion, and recurrence after 18 month has been presented in Table 3. The results of Chi-square Test indicated that there is significant statistical association between types of HPV and tumor grade (*P* = 0.044). However, there were no significant statistical association between sex, tumor stage, muscle invasion, and recurrence after 18 month (*P* > 0.05). We investigated the relationship between recurrence after 18 month with demographic and prognostic factors of bladder cancer patients using univariate and multivariate analysis (Table 4). Only variables with *P* < 0.2 in the univariate analysis were included in multivariable analysis. Hence, we report odds ratio and their 95% confidence intervals by logistic and penalized logistic regression tests. All analysis performed by STATA software ver. 13. Based on the results, family history of cancer showed a significant association with recurrence. The odds ratio of tumor recurrence within 18 months in patients with a family history of cancer is 4.05 times more than that of patients with no family history of cancer (*p* = 0.003). Moreover, there was a significant relation between HPV infection and recurrence within 18 months. The odds ratio of recurrence in patients with HPV infection is approximately 7 times more than those without HPV infection (*p* = 0.008). According to the univariate analysis results, a statistically significant relationship between recurrence and T3-T4 tumor stage compared to T0 tumor stage has been reported (*p* = 0.015). However, in multivariate analysis after considering the effect of other variables affecting recurrence, there was no significant relationship between recurrence and muscle invasion (*p* = 0.478).

**Table 3:** Association between HPV types and sex, tumor grade, tumor stage, muscle invasion, and Recurrence after 18 month

Variable	Types of HPV				***P***-Value
	Type 6 (%)	Type 11(%)	Type 16 (%)	Type 18(%)	
Sex					
**Male**	1 (4.80)	6 (28.60)	8 (38.10)	5 (23.80)	0.781
**Female**	0 (0)	0 (0)	2 (66.70)	1 (33.30)	
Tumor Grade					
**Low grade**	1 (14.30)	4 (57.10)	2 (28.60)	0 (0)	**0.044**
**High grade**	0 (0)	2 (11.80)	8 (47.10)	6 (35.30)	
Tumor Stage					
**Ta**	1 (20)	3 (60)	1 (20)	0 (0)	0.246
**T1**	0 (0)	1 (33.33)	1 (33.33)	1 (33.33)	
**T2**	0 (0)	2 (28.60)	2 (28.60)	2 (28.60)	
**T3-T4**	0 (0)	0 (0)	6 (66.70)	3 (33.30)	
Muscle Invasion					
**Positive**	0 (0)	2 (12.50)	8 (50)	5 (31.30)	0.133
**Negative**	1 (12.50)	4 (50)	2 (25)	1 (12.50)	
Recurrence after 18 months					
**Positive**	1 (4.20)	6 (25)	10 (41.70)	6 (25)	1.00
**Negative**	1 (4.20)	6 (25)	10 (41.70)	6 (25)

**Table 4:** Relationship between recurrence after 18 month and patient characteristics

Variable	Univariate analysis		Multivariable analysis	
	OR (95% CI)	***p***-value	OR (95% CI)^**a**^	***p***-value
**Age**	0.95 (0.915–0.992)	0.018	0.99 (0.94–1.04)	0.762
**Gender**				
Female	Reference		Reference	
Male	1.02 (0.39–2.68)	0.967	–	–
**Cigarette smoke**r				
Non-smoker	Reference		Reference	
Smoker	1.66 (0.67–4.09)	0.272	–	–
**Family history of tumor**				
Negative family history	Reference		Reference	
Positive family history	4.45 (1.90–10.41)	0.001	4.05 (1.61–10.19)	**0.003**
**HPV**				
Negative	Reference		Reference	
Positive	12.34 (3.13–48.57)	< 0.0001	6.93 (1.65–29.23)	**0.008**
**Tumor grade**				
Low grade	Reference		Reference	
High grade	2.44 (1.09–5.46)	0.030	0.95 (0.08–10.66)	0.961
**Tumor Stage**				
T0	Reference		Reference	
T1	1.31 (0.39–4.36)	0.665	1.09 (0.22–5.40)	0.911
T2	1.96 (0.79–4.85)	0.146	0.25 (0.01–9.33)	0.451
T3-T4	6.15 (1.42–26.72)	0.015	1.16 (0.16–8.19)	0.878
**Muscle invasion**				
Negative	Reference		Reference	
Positive	2.67 (1.16–6.11)	0.020	4.62 (0.07–0.317.93)	0.478

*OR Odds ratio, *CI* Confidence interval

## Discussion

Involvement of HPV in several kinds of cancers, including cervical, oropharyngeal, and anal cancers, have been reported in earlier studies [18]. HPV infection is spontaneously cleared in most cases. In the remaining instances that develop into persistent infections, particularly in infections with high-risk types of the virus, the risk of malignancy can increase [19]. The results of a meta-analysis that analyzed 52 studies comprising 2855 cases showed an incidence between 0 to 100% for HPV in bladder tumor samples [20]. This meta-analysis mentioned a role for HPV type 16 in the carcinogenesis of the bladder [20]. An earlier study in Iran reported a rate of 36.4% for HPV infection in TCC tumors [12]. In our study, HPV DNA was present in 22.6% of tumoral bladder tissue samples, mostly of HPV type 16, followed by types 11, 18, and 6.

It has been determined that the prevalence of HPV varies among different populations and geographical regions. Furthermore, diagnostic methods used in the detection of the virus affect the rates reported for its prevalence [21]. Another factor suggested to account for these differences in the prevalence reported for bladder tumors is that the virus might not infect all parts of the tumoral tissue equally. Thus, if the samples are not from the infected site, the test may yield a false-negative result. Likewise, contamination at the time of sampling may lead to false-positive results. These issues emphasize the importance of obtaining multiple samples [22].

All HPV positive samples in the present study were infected with a single type of the virus, and infection with multiple types was not observed. This result is not in agreement with the findings of an earlier study which has detected different types of HPV simultaneously in the sample and has stated that simultaneous infection with several types of HPV virus may increase the risk of carcinogenesis [22].

Even though the rate of bladder cancer is higher in men than in women [23], the results of our study suggest that there is no relationship between sex and the grade and stage of the bladder tumor or its recurrence (Table 2). In a study conducted in the past, the rate of HPV infection was higher in women than in men [24]. In that study, it was stated that the relative shortness of the urethra in women usually causes the rate of the urinary infection to be higher in them than in men and affords easier access for infectious agents to produce ascending infections and reach the bladder [24].

In earlier studies, a significant relationship was established between cigarette smoking and the risk of bladder cancer [25], but the results of our study did not show a significant relationship between smoking and the grade and stage of the tumor and its recurrence, or muscle invasion of the tumor.

The relationship between HPV infection and bladder cancer has been addressed in several previous studies [7, 26]. A study conducted in Iran showed that infection with HPV, particularly type 18, increased the risk of bladder cancer, but that study did not find any relationship between the infection with this virus and the grade of the tumor [7]. However, the results of the studies on the relationship between this virus and bladder cancer have been conflicting, and the results of some studies have not confirmed such a relationship [21]. The results of a previous study suggested an association between the high-risk types of HPV and low-grade bladder cancer [6]. However, a recent study that used fresh tissue samples for detecting HPV refuted a relationship between this virus and bladder tumor grade [27].

In general, the importance of HPV infection as a prognostic factor for the survival of urological cancer patients has been assessed in other studies [9, 28]. Several studies have also addressed the relationship between the grade of the bladder tumor and HPV infection [12]. Some studies found a higher prevalence of this virus in the low-grade tumors [22, 24], while some other studies found a relationship between the higher grades and stages of this tumor and HPV infection [12]. In the present study, HPV infection was found more in tumors in stages T3–4. In addition, the prevalence of HPV in low- and high-grade tumors was 29.2% and 70.8%, respectively. Thus, our findings indicate a relationship between HPV infection and the higher grade and higher stage of the tumor.

It has been shown that most of bladder tumors recur within 12 months of treatment [29]. The encounter with infectious agents has been considered as one of the risk factors for urologic malignancies, particularly in patients with a high tumor recurrence rate [22, 30]. In a study by Badawi et al., a significant relationship between the rate of tumor recurrence and the presence of HPV-16 and anti-HPV antibodies was revealed [22]. Furthermore, the authors stated that a significant relationship existed between the rate of the prevalence of HPV-16 and tumors in grades 1 and 2 and stage 3, as well as muscle invasion of the tumor [22]. The significant role of HPV in the progression of TCC tumors to higher stages and grades was discussed in another study by Youshya et al., and they named the inactivation of tumor suppressors by HPV as one of its possible causes [31].

Preliminary analysis of our data showed that HPV infection had an association with age, and the mean age of the HPV-positive individuals was 6 years less than those patients in which HPV was not diagnosed. This result is in agreement with the findings of a previous study [6]. However, some studies have stated that there is no association between HPV infection and age [22]. As HPV is a sexually transmitted virus, it is not unlikely that younger ages may show higher infection rates. The infection with this virus gains more importance because a relationship has been established between HPV infection and invasive cervical cancer [32], and men can transmit this virus to their sexual partners. This can also point to the possible importance of HPV vaccination in men [33, 34].

Previous studies have shown that there is a relationship between the grade of the bladder tumor and age and also that a higher rate of low-grade tumors is observed in the older ages [6], but no such relationship between the age and grade of the tumor was observed in our study. However, we found a significant relationship between age and recurrence rate and stage of the tumor, and those patients who had been affected by bladder cancer at a younger age showed higher recurrence rates and higher stages of the tumor. Assessment of the relationship between HPV types with tumor characteristics (stage, grade, and muscle invasion) and tumor recurrence showed a relationship between types 16 and 18 and the grade of the tumor.

In our study, a family history of cancer had an association with the grade of the tumor, muscle invasion, and tumor recurrence. Furthermore results of the multivariant analysis, after the adjustment of the variables, showed a significant relationship between the variables of family history and HPV infection with tumor recurrence within 18 months, while any significant relationships between the grade and stage and muscle invasion of the tumor and tumor recurrence rate was found. Thus, it can be concluded that a family history of cancer and HPV infection can be potential independent predictive factors for tumor recurrence in bladder cancer. Overall, the results of this study strongly indicate a significant relationship between HPV infection and an aggravated outcome of the disease and a higher risk of recurrence in patients with bladder cancer.

However, no study is without limitations. Our data was collected from only one center and although we included more than 100 cases, it is not a multi-center study. Secondly, the samples were in FFPE form and were not fresh frozen tissue (FFT). Quality of PCR might be slightly higher in FFT samples compared to FFPE; however, the difference is trivial and non-significant in many cases [35, 36]. For instance a previous study declared the superiority of fresh tissue samples over FFPE [27]. However, difficulties in acquiring and maintaining FFT samples results in much more common use of FFPE [37].

## Conclusions

Bladder cancer, especially its muscle invasive form has a poor prognosis and high probability of recurrence, and studies have shown a mean survival rate of about 28 months for the patients who experience bladder cancer recurrence [38]. There is no main etiologic factor in the occurrence of this cancer and its oncologic consequences, therefore, the identification of an infectious viral factor can be regarded as a possible element for the prevention and treatment of this disease. Thus, it seems necessary to conduct case-control studies on large numbers of cases to investigate the effect of HPV infection on bladder cancer development and its outcomes. Moreover, the direct role of this virus in carcinogenesis in the bladder should be further studied. Likewise, because of the sexual transmission of this virus and its important role in carcinogenesis and oncologic consequences of cancer, the vaccination policy for both sexes appears worth contemplating and merits further review.

## Data Availability

All data used and/or analyzed during the present study are included in this manuscript and also available from the corresponding author.

## Abbreviations

FFPE: Formalin fixed-paraffin embedded
HPV: Human papilloma virus
PCR: Polymerase chain reaction
STIs: Sexually transmitted infections
TCC: Transitional cell carcinoma
WHO: World Health Organization
